# Vegan Egg: A Future-Proof Food Ingredient?

**DOI:** 10.3390/foods11020161

**Published:** 2022-01-08

**Authors:** Fatma Boukid, Mohammed Gagaoua

**Affiliations:** 1 Food Safety and Functionality Programme, Institute of Agriculture and Food Research and Technology (IRTA), 17121 Monells, Spain; fatma.boukid@irta.cat; 2 Food Quality and Sensory Science Department, Teagasc Food Research Centre, Ashtown, D15 KN3K Dublin, Ireland

**Keywords:** future foods, sustainability, egg, algae, starches, plant proteins, bakery products, mayonnaise, food formulation

## Abstract

Vegan eggs are designed with the aim to provide a healthier and more sustainable alternative to regular eggs. The major drivers of this industry are the increasing prevalence of egg allergies, awareness towards environmental sustainability, and the shift to vegan diets. This study intends to discuss, for the first time, the vegan egg market, including their formulation, nutritional aspects, and some applications (i.e., mayonnaise and bakery products). Recreating the complete functionality of eggs using plant-based ingredients is very challenging due to the complexity of eggs. Current, but scarce, research in this field is focused on making mixtures of plant-based ingredients to fit specific food formulations. Nutritionally, providing vegan eggs with similar or higher nutritional value to that of eggs can be of relevance to attract health-conscious consumers. Claims such as clean labels, natural, vegan, animal-free, gluten-free, and/or cholesterol-free can further boost the position of vegan eggs in the market in the coming year. At present, this market is still in its infancy stages, and clear regulations of labeling, safety, and risk assessment are deemed mandatory to organize the sector, and protect consumers.

## 1. Introduction

Eggs play significant roles in foods, owing to its gelling, foaming, and emulsification features [1,2]. Eggs are versatile products available in the market in dry and liquid forms as whole eggs, egg whites, and egg yolks. Besides their functionality, eggs are of particular interest from a nutritional point of view since they contain proteins, vitamins, minerals, essential fatty acids, and other micronutrients [3]. These components are distributed between the egg yolk and egg white. Egg yolk is rich in lipids (65–70% on dry basis) and proteins (30% on dry basis), and it is a good source of lutein, zeaxanthin, and vitamins [4]. Egg white is rich in proteins, namely fibrous structural proteins (ovomucins), glycoproteins (ovalbumin, protease inhibitors), antibacterial proteins (lysozyme), and peptides [3].

Despite the nutritional value and functionality of eggs as a food ingredient, a high consumption was reported to be related to chronic degenerative diseases that can cause cardiovascular disease and mortality due to its high cholesterol content [5,6]. Moderate egg consumption (up to one egg per day) was found to be not associated with a cardiovascular disease risk [5]. Overall, it was recommended to limit cholesterol intake, and replace whole eggs with egg whites/substitutes for facilitating cardiovascular health and long-term survival [7]. In low- and middle-income countries, due to the price increase in meat, there has also been a shift towards increased egg consumption as a response to maintaining animal-based protein consumption in the diet [8]. On the other hand, consumers are turning towards plant-based food substitutes as a response to rising food safety concerns related to eggs, among other ethical concerns in European countries such as Germany, France, and Italy [9,10,11]. Zoonotic diseases related to poultry and eggs, such as avian tuberculosis, erysipelas, and salmonellosis, and flus, is one of the main factors fueling the market growth of vegan eggs [12]. Over the last years, increased public awareness over numerous foodborne diseases transmittable through egg consumption are increasingly alarming for consumers, and negatively impacting the consumption of poultry eggs [13]. Several safety scandals have heavily affected the egg industry, such as fipronil in eggs in Europe (2017), and a salmonella outbreak in poultry in the USA (2015) and UK (2018) [13,14]. The use of antibiotics and hormones in poultry products to ensure rapid growth and health is another contentious issue in the poultry industry [15]. Nevertheless, the accumulation of these drug residues in eggs can cause significant health concerns by triggering allergic reactions and transmitting antibiotic-resistant microbial infections [16]. Egg allergies are one of the most common food allergies in young children, and tends to persist into adulthood [17,18]. This allergy is triggered by ovomucoid (Gal d 1), ovalbumin (Gal d 2), ovotransferrin/conalbumin (Gal d 3), and lysozyme (Gal d 4), which are mainly located in egg whites and, to a lesser extent, in yolk [19,20]. This allergy can cause serious skin reactions, nasal congestion, and gastrointestinal and respiratory symptoms [21]. Egg allergies may also coexist with other food allergies, such as nuts and fish [22,23]. A diet exempt of egg is the only solution to manage an egg allergy [24]. Different approaches were applied to reduce cholesterol and to mitigate epitopes present in eggs by chemical (solvents and biopolymers), physical (fractionation and separation), and biological (enzymes) processing [25,26]. These methods showed variable degrees of success, but they have not been scaled up due to safety, consumer acceptance, repeatability, and economic reasons [27]. These issues have increased consumers’ concern toward the consumption of eggs for health, safety, or environmental reasons, and gave room to a new variety of alternative products, such as egg substitutes. 

Creating egg substitutes to replace egg functionality and nutritional properties is a challenging task due to the multi-functionality of eggs that impact the taste, texture, and the aspect of food products. The first studies on egg substitutes go back to the seventies [28,29], where particular focus was attributed to replace eggs in bakery products. At first, animal proteins were mostly used, such as milk powder, casein, whey, and bovine plasma protein [30,31]. This is due to their excellent functional properties, such as solubility, emulsification, foaming, and heat-induced gelation properties [32]. Despite the occurrence of animal ingredients in the human diet, plant-based foods are gaining popularity around the world due to their health benefits, environmental sustainability, and ethical merit [33,34]. The coronavirus (COVID-19) outbreak consolidated this transition due to changes in consumers’ dietary habits, associating plant-based diets to be healthier alternatives to animal products [35]. COVID-19 caused also a decrease in the demand for chickens and eggs, resulting in prices fluctuations due to lockdown restrictions limiting business opportunities and customer incomes [36]. Furthermore, there are false rumors suggesting zoonotic origins of COVID-19 or poultry products contributing to the spread of COVID-19 [12,37]. 

To cater for the growing vegan and health-conscious market, manufacturers have created vegan eggs using different types of plant-based ingredients (e.g., proteins, polysaccharide hydrocolloids, or emulsifiers), alone or combined, to replace regular eggs in food products. These ingredients might present nutritional benefits such as low allergenicity, reduced price, and high production volumes. Nevertheless, the functional properties are highly variable among the different ingredients in terms of composition, purity, and source [38]. The vegan egg market keeps growing to deliver different products with different properties to fit a wide range of applications. In this context, this study aimed, for the first time, to: (i) enable an overview about the current market landscape of vegan eggs, with a focus on drivers and barriers; (ii) address the main ingredients used in formulating vegan eggs; (iii) discuss their nutritional properties in comparison to conventional egg products; and (iv) confer their impact on food products, with a focus on mayonnaise and bakery products as examples among other potential products. 

## 2. Global Market Landscape of Vegan Eggs

The global vegan egg market is moving upward, and accounted for US$1.5 billion in 2021, and is expected to witness a high compound annual growth rate of 8.3% through 2031 [39]. Due to the absence of exhaustive market reports about vegan eggs launched in the global market, the authors made their search using Mintel’s GNPD database [40], with a focus on the period 2016–2021 to capture the current market landscape of vegan eggs. From 2016 to 2021, 102 vegan egg products were launched in the global market. The authors gathered all information on the front-of-pack labelling. Table 1 summarizes the main market trends in the vegan egg industry, relying on the main claims used on the retrieved products. Health and well-being, naturalness, sustainability, and convenience are the main trends, with “vegan/no animal” and “vegetarian” ingredients (related to health and well-being and sustainability), and “low/no/reduced allergen” and “gluten-free” (related to health and well-being) being the top four sub-trends [40]. In the last decade, consumers have become more concerned on health and well-being, and are paying more attention to what they eat. As a result, manufactures of vegan eggs consider the use of a large spectrum of ingredients to offer a portfolio of products to accommodate all consumers, including those with special needs. Indeed, 80.4% of marketed vegan eggs claim to have low/no/reduced allergens, including 65.7% and 18.6% claiming to be gluten-free and low/no/reduced lactose, respectively. Increasing niches with particular lifestyles, such as vegan, vegetarian, and flexitarian, contributed to the reduction of animal-derived products, such as alternative meat, vegan dairy, and vegan eggs [41,42,43]. In addition, 100% claim to be suitable for vegans and vegetarians due the absence of animal-based ingredients in egg formulations, such as whey protein, milk, or casein. Emphasizing that these products are made with plant-based ingredients was reflected by the use of term “plant-based” on 31.4% of products. This shift to non-animal ingredients seemed to continue to benefit the industry of vegan eggs [33,44]. 

Vegan eggs are also rising as a healthier alternative to eggs, since they contain no cholesterol. This aligns with market trends reporting around 41.2% of launches claimed to have low/no/reduced cholesterol. This industry is further focused on designing products with reduced sugar, fat, saturated fat, and sodium. Since consumers have a strong preference for food products free from additives and preservatives, there is a growing trend boosting the use of natural and clean label ingredients [45]. This was reflected by declarations, such as genetically modified organisms (GMO)-free (35.3%), organic (27.4%), no additives/preservatives (22.5%), free from added/artificial preservatives (7.8%), and free from added/artificial colorings (5.9%). Consumers’ awareness towards contaminants is also considered where terms like “toxin-free” were used to describe 1.96% of the products. Convenience is an important driver of this market, in which 34.3% of products were declared as easily used. This aligns with a general trend in the food sector seeking quick and convenient meal solutions [46]. Finally, sustainability is becoming an essential criterion in the food sector [47], and the sustainability of vegan eggs is reflected by the fact that 83.3% of the products seemed to have environmental or ethical claims, including recycling food waste, and the use of sustainable packaging. 

Like other emerging alternative products, the current market barriers of vegan eggs are the lack of high production volumes, targeted marketing, and clear regulations. A recent study based on in-depth interviews with egg industries and retailers and plant-based egg manufacturers revealed that replicating all eggs’ nutrients and functionalities is not realistic, and considering plant-based eggs as potential competitors to conventional products is impossible. Also, there is uncertainty on how to present the labeling of plant-based eggs [48]. Consumer perception and acceptance is also an important factor for the growth of such a novel food sector. Consumer expectations from vegan egg products were found to be depending on product-related (color, shape, taste, ingredients, nutrients, method of production, and shelf life) and non-product-related attributes (price, packaging, country of origin, and product naming) [49]. More in-depth quantitative and quantitative studies are required for a deeper understanding of this first screening based on country surveys. From a manufacturer perspective, the main challenge of vegan eggs can be related to the difficulty in delivering similar nutrition, taste, and functionality to eggs [48].

Considering the current global market landscape (Table 2), vegan eggs are marketed in different forms (powder, liquid, and egg-shaped) [39]. The powder segment is the most dominant, and was estimated to be US$815.4 million in 2019 [50]. The demand of powder vegan eggs is expected to keep increasing at a high rate, with the on-the-go nature or ease of use claims establishing these products as convenient healthy snacks [51]. The most sold vegan eggs are made from starches, plant-based proteins, soy products (lecithin, tofu, and tahini), algae flours, and other ingredients (e.g., fruit purees and vinegar) [39,50]. By region, North America is estimated to account for 47.8% of the global market, and is expected to remain the dominant one until 2026 [50]. By the end of 2021, sales of vegan eggs in North America are expected to reach US$476.6 million, corresponding to 32% of global sales [39]. The vegan eggs market is trending in Europe due to their applications in reformulating snacks and meat alternatives [39]. The most important producers of vegan eggs are Corbion NV (Amsterdam, The Netherlands), Glanbia Plc (Kilkenny, Ireland), Tate & Lyle Plc (London, UK), Ingredion Incorporated (Westchester, IL, USA), Ener-G Foods, Inc. (Seattle, WA, USA), Natural Products, Inc. (Grinnell, IA, USA), Orchard Valley Foods Limited (Tenbury Wells, UK), Puratos Group (Dilbeck, Belgium), TerraVia Holdings, Inc. (San Francisco, CA, USA), and Archer Daniels Midland Company (Chicago, IL, USA) [50]. Vegan eggs are mostly used as substitutes of eggs in bakery products, desserts, and confectionary [39]. The mayonnaise segment is estimated to account for a value share of 38.2% in the global market, whereas bakery products are estimated to account for over 26% [50]. Claims such as natural, organic, clean label, Halal or Kosher certified, dairy-free, GMO-free, and gluten-free are also boosting the market of vegan eggs [50]. 

## 3. Major Components of Vegan Eggs

Vegan eggs can be formulated by one plant-based ingredient or a combination of ingredients to recreate the functionality of eggs Pulses are ingredients rich in proteins, starches, and fibers, as well several health beneficial ingredients [52]. Proteins deriving from pea, lentil, lupine, and chickpea can confer in their native and modified forms interesting functionalities, such as gelling, emulsification, and foaming for formulating vegan eggs [53,54,55,56]. The proteins can be used in different forms, namely flours, protein concentrate, or isolates. Besides their high nutritional value, pulses are known for their affordability and sustainability [52]. Furthermore, pulses are recognizable products by the consumers, and their inclusion in vegan egg formulations might contribute to their acceptability. The proteins of pulses have plenty of pros, but they have some nutritional limitations, such as their low content in sulfur amino acids, which can be overcome by blending them with cereals. Also, plant proteins have a globular structure that impacts the functionality and, more specifically, the solubility. To overcome such concerns, the addition of hydrocolloids was suggested to improve the functionality of proteins [57,58]. As an alternative, these proteins can be improved by postprocessing using thermal treatments, fermentation, and crosslinking by means of enzymes to improve the emulsification, gelling, and foaming abilities [59,60,61]. Pulses also present flavors described as “beany” or “green”, attributed to their content in saponins, ketones, and aldehyde compounds [62]. Several solutions are being applied to attenuate these flavors, such as using masking agents and mitigation processing [63,64]. Starches from pulses are also increasingly used in formulating vegan eggs to play the role of binding and thickening [65]. Native starches from pulses have some functional limitations compared to those usually used, such as tapioca and corn starches [66,67]. Nevertheless, several postprocessing methods are being developed to produce modified starch with high quality, likely-modified pea starch [68,69]. Another ingredient, aquafaba, derived from cooked chickpea, is gaining interest as an egg substitute due to its foaming, emulsifying, thickening, and gelling properties [70,71,72]. This is attributed to its composition, namely protein, water-soluble/insoluble carbohydrates, coacervates, saponins, and phenolic compounds [62,73]. The main limitation for the commercialization of aquafaba is the lack of product standardization due to the high variability in chickpea properties (differences in the composition and genotypes) and processing conditions (temperature, pressure, and cooking time) [38,74,75,76].

Different types of hydrocolloids, such as carrageenan, pectin, and guar gum, have been used as natural foaming, thickening, and emulsifier agents to further reinforce the structure made by plant-based proteins and starches, and for an improved mouthfeel [77,78,79]. Fibers from pulses are also of relevance in vegan egg formulations due to their gelling, binding, and thickening properties. Nevertheless, the most used fibers derive from apple, citrus, and oat fibers. Cellulose derivatives, such as carboxymethyl cellulose or hydroxypropyl methylcellulose, can be used as thickeners or emulsifiers. 

Oilseeds (mainly soybeans) are also used in different forms, such as proteins, flour, or milk, owing to their high protein content, complete essential amino acids, and protein digestibility that can be comparable to that of animal proteins [33]. In recent years, consumers have been concerned about soy ingredients for their genetically modified reputation and allergenicity [80,81]. This has given room for more emerging sources, such as oat, mung bean, lentil, and faba bean [82,83,84]. 

Emerging ingredients, such as algal flours, are also of interest as food ingredients due to their high nutritional quality and sustainability [85]. They are a rich source of proteins, lipids, fibers, and vitamins [86]. Compared to plant ingredients, algae are also a good source of vitamin B12 for vegetarians and vegans [87]. They also contain functional ingredients, such as monoglycerides, diglycerides, and phospholipids, mainly acting as emulsifiers [88,89]. Indeed, the first vegan egg (VeganEgg) using algal flours was launched in 2017. 

Vegetable oils, such as canola and sunflower oils, are also important as structuring agents in vegan egg formulations, hence contributing to the creation of the textural attributes, flavor profile, and mouthfeel of the final products [70]. Flavoring agents such as Himalayan black salt or “Kala namak” are commercially available to mimic the sulfur flavor of egg [90]. Other ingredients can be added, such as spices (e.g., garlic powder, sugar, and salt), buffers (e.g., bicarbonates or phosphates), and preservatives (e.g., nisin) [47].

## 4. Nutritional Value of Vegan Eggs

This section provides an overview of the nutritional composition of vegan eggs, yolks, whites, and whole eggs launched in the global market from January 2016 to October 2021 (Table 3). Based on Mintel’s GNPD database, 102 new vegan egg products were launched to the global market [40]. The major ingredients in egg products are proteins and fat, whereas vegan eggs have a profile rich in carbohydrates, proteins, and fibers. Vegan eggs provide the highest calories, followed by whole eggs, yolks, and egg whites. This is due to their high content in carbohydrates (41.89 g/100 g), in which starch content (66.73 g/100 g) is the main contributor due to starchy ingredients (in the form of starchers and flours) used in vegan products. Egg carbohydrates were found mostly in egg yolks, whereas a lower amount was found in egg whites and whole eggs. It was reported that glucose is the dominant free sugar in the eggs, with traces of fructose, lactose, maltose, and galactose [91]. Total fat and saturated fat contents were found lower in vegan eggs compared to the whole egg and yolk, but higher than the egg white. This can be attributed to the use of vegetable oils rich in saturated fats, such as palm oil. Noteworthy, vegan eggs are cholesterol-free, whereas whole eggs have the highest value, followed by egg yolks, and egg whites. The whole egg and yolk have the high cholesterol content, exceeding the limits set by the American Heart Association of <300 mg/day [92].

Eggs do not contain any fibers. However, vegan eggs provide high amounts of fibers that are added to mimic the emulsification properties of eggs. Egg whites and egg yolks are almost equality concentrated in proteins, but slightly higher than vegan eggs and whole eggs. Vegan eggs are made with different proteins to reach similar content to that of the conventional product, but little is known about their amino acid profiles. This underlines the great efforts being made to have a similar protein content to animal counterparts, which is usually known as a limitation of vegan products, including meat and dairy alternatives [41,43]. Vegetable proteins are the most used sources for compensating the protein content reduced by egg removal. It is well-known that animal proteins have a complete composition of essential amino acids and high digestibility compared to plant-based products [33]. It will be of interest to investigate such parameters in vegan products to address it in future product development projects. 

Sodium was found to be higher in vegan eggs compared to regular eggs, egg yolks, and egg whites. A lower amount of sodium was previously reported in whole eggs (142 mg per 100 g of whole egg) [91]. This can be attributed to the increase of yolk-to-egg-white ratio [3,91]. Vitamin B12 is a big limitation in vegan eggs compared to whole eggs. For these reasons, fortifying vegan egg products with bioavailable forms of these micronutrients is required [93]. However, the nutritional facts of commercial yolks and whites did not present the amounts of B12, since it is not mandatory information. Surprisingly, calcium was found the highest in vegan eggs, showing the direction in new product development focusing on upgrading the nutritional value of vegan products.

## 5. Main Food Applications of Vegan Eggs

### 5.1. Egg-Free and Egg-Reduced Mayonnaise

Mayonnaise is one of the most popular condiments worldwide, providing a creamy texture and special flavor [94]. Mayonnaise is a colloidal system (oil-in-water emulsion) made from vegetable oil (70–80%), egg yolk, vinegar, salt, and spices [95]. Egg yolk is a key ingredient for emulsion stability due to its high emulsifying capacity attributed to the phospholipids and lipoproteins (high-density lipoprotein and low-density lipoprotein), and non-bonded proteins (phosvitin and livetin) [96]. Egg yolk also provides forming properties and prevents flocculation to ensure an appropriate texture of mayonnaise [1,97]. Nevertheless, the use of raw eggs in mayonnaise might present some inconveniences, such as possible contamination with Salmonella sp., and high cholesterol content [98]. As an alternative, egg-free mayonnaise is gaining traction as a healthier option for consumers, and is suitable for vegan customers, as well as being more cost-effective (no pasteurization is required). Several vegan eggs were used in single and combined forms to mimic the quality, taste, and color of conventional mayonnaise [98]. 

Vegetable protein isolates deriving from soy, pea, lentil, and rapeseed have been considered as suitable egg alternatives [33,99,100]. Egg-free mayonnaise designed using 6% soy protein concentrate (as an emulsifier to replace egg yolk) was accepted by consumers [101]. A mayonnaise was made with a 10% substitution level of eggs, using a vegan egg made by a combination of soy milk and a blend of 6.7% mono- and di-glycerides, 36.7% guar gum, and 56.7% xanthan gum. This low substitution level produced a low cholesterol-low fat mayonnaise with improved properties (i.e., the stability, heat stability, consistency coefficient, viscosity, firmness, adhesiveness, adhesive force, and overall acceptance) [102]. Eggs were replaced with soy milk at levels of 25, 50, 75, and 100%. Results showed that up to a 75% egg substitution level, viscosity was not affected, whereas stability was decreased. The sensory acceptability of the products was not impacted until 50% substitution level. This suggests that soy milk can be a good candidate to partially substitute egg (up to 50%) without hampering product viscosity and taste [103]. Nevertheless, combining soy milk with different hydrocolloids (i.e., xanthan gum and zodo gum) increased in the apparent viscosity, the consistency coefficient, and the firmness/emulsion stability of the mayonnaise, whereas the mayonnaise flow index was reduced. The optimal formulation of vegan eggs was 0.25% xanthan gum, 3.84% zodo gum, 37.50% oil, and 63.61% soy milk [104]. Egg yolk replaced with sesame-peanut meal milk decreased product quality, including pH, color, thermal stability, and acidity, with increasing substitution levels (0, 25, 50, 75, and 100%). Mayonnaise made with vegan eggs at 50% had desirable physical and thermal stability, and reduced cholesterol content [105].

Raikos et al. [106] reported that the use of liquid aquafaba (up to 70%) was capable of forming a stable emulsion resulting in mayonnaise with a desirable consistency and unaffected oxidative stability during storage. Using dry aquafaba resulted in a high stable mayonnaise for 28 days of storage at 4 °C. These results suggest that the use of dry aquafaba can be the solution to overcome standardization issues of aquafaba, and can be effectively used in mayonnaise manufacturing [75].

Starches were also used as ingredients to replace egg yolk in mayonnaise formulation, owing to their thickening properties. Native starches were found undesirable due to their unfavorable effect on the texture and flavor. Modified starches, such as octenyl succinic anhydride-modified potato starch, showed better emulsification properties when partially replacing eggs (0, 25, 50, 75, and 100%). Products made with 75% octenyl succinic anhydride-modified potato starch resulted in high emulsion stability even after two months of storage, and it also reduced cholesterol content, improved oil droplets particle size (maximum at 70 µm), and resulted in a consistent texture with no agglomerates. This is due to the formation of a stable cohesive layer of starch surrounding the oil droplets [97]. Mayonnaises were prepared with 35% freeze-dried chia mucilage instead of egg yolk due to their emulsification properties [107,108]. The resulting mayonnaise had similar stability and texture parameters, as well as sensory acceptance, to the control mayonnaise [109]. 

Several thickeners, such as gums (xanthan and guar gums, and Arabic gum), were also used for egg reduction, or complete removal, due to their emulsifying ability and stability [110]. Durian seed gum used at a level of 4% resulted in vegan mayonnaise with textural and sensory properties comparable to egg-based products [111]. As such, this substitution was able to generate a stable emulsion, and to prevent coalescence and flocculation for prolonged periods of storage (up to 5 months). Arabic gum may have inhibitory effects towards lipid oxidation and microbial contamination, owing to its high antioxidant activity [98]. Overall, these hydrocolloids improve emulsification, antibacterial activity, and sensory quality of the final product [110], whereas guar gum and/or xanthan are considered additives that are not fully appreciated by consumers seeking “clean” labels [94].

Algal ingredients, such as *Chlorella vulgaris*, were also used in partially substituted yolk in combination with acid casein curd. A mix of *Chlorella vulgaris* (10 and 15%) and casein curd (90 and 95%) improved the nutritional value, rheological properties, and sensory scores of mayonnaise at 25 and 50% of egg replacement [112].

### 5.2. Egg-Free and Egg-Reduced Bakery Products

Egg exclusion or reduction comes in the optic to promote the healthiness of egg-free and/or cholesterol-free bakery products. The total substitution of eggs by lupine protein isolates resulted in the collapse of the cakes. This can be explained by the lower functional properties compared to egg [54]. Thus, besides lupine isolates, soy lecithin, mono- and diglycerides, and xanthan gum were used as vegan egg substitutes. The resulting cakes had an improved structure of crumb, reduced shrinkage, and led to high height [113]. Likewise, a blend of soy protein isolate and 1% mono- and di-glycerides produced an egg-free cake with similar specific volume and gravity, firmness, and moisture content compared with egg-containing cakes [114]. Similarly, the use of only soymilk to replace eggs resulted in a batter with low density and viscosity, resulting in a firm, dark, and compact cake, whereas combining soymilk and soy lecithin improved the quality of egg-free cakes [115]. In another study, egg-free and egg-less cakes were successfully produced by replacing eggs with a mix of lupine protein, whey proteins, and soy lecithin [116]. The complete substitution of egg whites by *Chlorella vulgaris* decreased the consistency of the batter, which imparted the cake with low specific volume, and a high weight loss and hardness. Nevertheless, a partial substitution level (25%) did not affect the taste, color, odor, texture, and overall acceptability compared to the conventional preparation [117]. Aquafaba-based cakes resulted in a similar color and texture, as well sensory acceptability compared to egg-white-based cakes. This is due to the good foaming and emulsifying properties of aquafaba [38,70,71]. The main defect of these eggless cakes is their low springiness and cohesiveness [74]. A potential approach might be adding other ingredients together with aquafaba to overcome this defect.

Egg-free and egg-reduced cakes were also produced through the complete use of hydrocolloids, such as hydroxypropyl methylcellulose in combination with sodium stearoyl lactylate [118,119]. Depending on the level of substitution and the type of additives, cake attributes, including color, texture, and volume, significantly changed, but in some cases, such changes were not perceived at the sensory level [115]. Nevertheless, this type of substitution is decreasingly desired due to the market shift towards natural ingredients. 

## 6. Conclusions

The market of vegan eggs is steadily growing as healthier, more sustainable, and ethical alternatives to regular eggs. Affordable and available ingredients are required to develop cost-effective vegan eggs. Although there is plenty of ingredients that can mimic the functionality of eggs, the nutritional value of vegan eggs must be carefully considered. Protein rich ingredients and vitamin/minerals fortification(s) are required to avoid nutritional deficiencies, especially in the case of vegan consumers. Natural and clean label ingredients are becoming a must by health-conscious consumers. There is no vegan egg fitting all food formulations. Therefore, the selection of egg replacements needs to be made based on the functionality required for each food product. At present, the market for vegan eggs is still a new commodity where clear regulation is required to organize the sector. Furthermore, in-depth market studies are required to capture this emerging market’s challenges and opportunities. Qualitative and quantitative surveys considering different countries, continents, gender, age, education level, and income are for interest to understand consumers’ behaviors toward such a new market. Consumer studies are needed to evaluate the sensorial properties of vegan eggs (different formulations) in comparison to regular eggs to provide a further understanding of the preferences and acceptability of consumers. 

## Figures and Tables

**Table 1:** Current trends in vegan eggs launched in the global market (2016–2021) ^1^.

Trends	Sub-Trends	Number of Products	Percentage Products Out of Total Launches (%)
Health and well-being	Minus		
	Low/no/reduced fat	4	3.92%
	Low/no/reduced trans-fat	1	0.98%
	Low/no/reduced sodium	4	3.92%
	Low/no/reduced calorie	2	1.96%
	Low/no/reduced cholesterol	42	41.2%
	Sugar free	3	2.94%
	No added sugar	3	2.94%
	Low/no/reduced saturated fat	1	0.98%
	Plus		
	High/added protein	5	4.90%
	Vitamin/mineral fortified	1	0.98%
	High/added fiber	8	7.84%
	Free from		
	Hormone free	2	1.96%
	Dairy free	39	38.20%
	Functional		
	Functional—other	1	0.98%
	Functional—digestive	1	0.98%
	Suitability		
	Low/no/reduced allergen	82	80.44%
	Gluten free	67	65.69%
	Kosher	43	42.16%
	Low/no/reduced lactose	19	18.63%
	Suitable for vegan and vegetarian	102	100%
	Plant based	32	31.37%
Convenience	Microwaveable	4	3.92%
	Ease of use	35	34.31%
	Convenient packaging	3	2.94%
	Time/speed	1	0.98%
Naturalness	No additives/preservatives	23	22.55%
	Free from added/artificial preservatives	8	7.84%
	Organic	28	27.45%
	Free from added/artificial colorings	6	5.88%
	GMO-free	36	35.29%
	Free from added/artificial flavorings	6	5.88%
	Natural product	3	2.94%
	Wholegrain	1	0.98%
	Free from added/artificial additives	3	2.94%
Ethical & environmental	Environmentally friendly package	33	32.35%
	Recycling	26	25.49%
	Sustainable (habitat/resources)	6	5.88%
	Environmentally friendly	8	7.84%
	Animal welfare	6	5.88%
	Toxins free	2	1.96%
	Biodegradable packaging	4	3.92%

^1^ Data based on Mintel’s GNPD database [40]. The query was conducted on 11 November 2021, and retrieved 102 vegan egg products in the global market from January 2016 to October 2021.

**Table 2:** Segmentation of the global market of vegan eggs adapted from [39,50].

Segment	Segmentation
Form	Powder Liquid Egg shape
Type	Starch Soy products (lecithin, tofu, and tahini) Plant proteins, such as pea and chickpea Algal flour Others (fruit purees and vinegar)
Application	Mayonnaise Biscuits and Cookies Cakes/Pastries/Muffins/Breads Chocolates Noodles and Pasta
Main players	Glanbia plc Ingredion Incorporated Cargill Bob’s Red Mill Natural Foods, Inc. House Foods America Corporation EVO Foods Mantiqueira (N.Ovo) JUST Inc. Orgran Foods Terra Vegane Free and Easy Follow Your Heart The Vegg Vezlay Foods Private Limited Now Foods The Neat Egg Conagra Brands, Inc. Ener-G
Region	North America Latin America Europe, Middle East, and Africa Asia Pacific

**Table 3:** Nutritional composition of eggs (per 100 g) and their alternatives in the global market ^1^.

	Vegan Egg	Egg Yolk	Egg White	Whole Egg
Number of retrieved products	102	37	54	6517
Average values of nutrients				
Energy (kcal/100 g)	298.55	153.66	98.36	152.18
Fat (g/100 g)	6.10	10.40	2.35	9.97
Of which saturated (g/100 g)	2.10	2.72	1.19	3.26
Carbohydrates (g/100 g)	41.89	3.77	2.59	2.32
Of which sugars (g/100 g)	1.77	3.77	0.53	0.45
Fiber (g/100 g)	8.56	0.00	0.00	0.00
Protein (g/100 g)	11.60	13.69	16.53	12.39
Sodium (mg/100 g)	912.59	682.67	353.01	385.74
Vitamin B12 (µg per 100 g/mL)	0.75	nr	nr	21,844.4
Cholesterol (mg per 100 g/mL)	0.00	339.26	11.64	1509.53
Calcium (mg per 100 g/mL)	286.59	39.88	122.23	159.80

^1^ Data based on Mintel’s GNPD database [40]. The query was conducted on 11 November 2021, and retrieved egg products launched in the global market from January 2016 to October 2021. nr: not reported.

## Data Availability

Not applicable.
